# Developing intelligent medical image modality classification system using deep transfer learning and LDA

**DOI:** 10.1038/s41598-020-69813-2

**Published:** 2020-07-30

**Authors:** Mehdi Hassan, Safdar Ali, Hani Alquhayz, Khushbakht Safdar

**Affiliations:** 1 Department of Computer Science, Air University, PAF Complex Sector E-9, Islamabad, Pakistan; 2 Directorate General National Repository, Islamabad, Pakistan; 3 Department of Computer Science and Information, College of Science in Zulfi, Majmaah University, Al-Majmaah, 11952 Saudi Arabia; 4 Al Nafees Medical College and Teaching Hospital, ISRA University, Lehtrar Road, Islamabad, Pakistan

**Keywords:** Machine learning, Computer science

## Abstract

Rapid advancement in imaging technology generates an enormous amount of heterogeneous medical data for disease diagnosis and rehabilitation process. Radiologists may require related clinical cases from medical archives for analysis and disease diagnosis. It is challenging to retrieve the associated clinical cases automatically, efficiently and accurately from the substantial medical image archive due to diversity in diseases and imaging modalities. We proposed an efficient and accurate approach for medical image modality classification that can used for retrieval of clinical cases from large medical repositories. The proposed approach is developed using transfer learning concept with pre-trained ResNet50 Deep learning model for optimized features extraction followed by linear discriminant analysis classification (TLRN-LDA). Extensive experiments are performed on challenging standard benchmark ImageCLEF-2012 dataset of 31 classes. The developed approach yields improved average classification accuracy of 87.91%, which is higher up-to 10% compared to the state-of-the-art approaches on the same dataset. Moreover, hand-crafted features are extracted for comparison. Performance of TLRN-LDA system demonstrates the effectiveness over state-of-the-art systems. The developed approach may be deployed to diagnostic centers to assist the practitioners for accurate and efficient clinical case retrieval and disease diagnosis.

## Introduction

Medical images are wellspring of learning on human life structures. These images are used to make visual illustrations of internal human body structure and pattern discovery for several types of clinical diagnosis such as brain tumor, breast, lung, and liver cancers. Generally, medical images are used for identification of particular aspect of affected tissue types and organs. Owing to human disease diversity and imaging modalities, it is challenging to classify the medical images compared to non-clinical images. The visual features of medical images are usually separated by subtle variation. For efficient and accurate disease diagnosis, radiologist requires several related clinical cases for analysis and interpretation of particular imaging modality. These modality images may also be used for teaching and demonstration purposes. Traditionally, specific modality images are retrieved manually from archives that is cumbersome and time-consuming processes. Manual annotation for retrieval is subjective and it does not represent image content properly, consequently it may mislead radiologist while analyzing new clinical cases. Archived medical image retrieval is critical due to the high cost of manual content annotation for tremendous image collections, variations in spellings, synonyms, and hyponyms^1^. Moreover, these images are acquired in different conditions, and associated information may be insufficient for interpretation and analysis^2,3^. The objective of a practitioner is to diagnose a disease based on similar archived clinical cases by employing the prior information and knowledge^4^.

Medical image archived databases provide cost-effective storage and convenient way compared to conventional text-based access over standard Picture Archiving and Communication Systems (PACS)^5,6^. Header of digital imaging and communication systems (DICOM) holds tags to interpret the observed body part and its modality^7^. This system automatically sets some of the tags as per the imaging protocol which is used to acquire the object image. Other tags are set manually by the medical expert/radiologists during routine documentation. This procedure is frequent and susceptible to error because some entries are either missed or not described automatically and precisely^8^.

Variety of imaging instruments are in practice to scan human body for disease diagnosis. For a clinician, modality is an essential characteristic to analyze human body anatomy and related clinical cases for an accurate diagnosis. Medical imaging archives are characteristically comprised of several types of modalities such as ultrasound, MRI, CT scan, PET, X-rays, etc. Retrieval technologies such as *Yottalook* and *GoldMiner* offering modality search facility, however modality information obtained by the image caption is annotated by the domain expert. Studies revealed that visual features might be useful for modality retrieval^9^. Several issues associated with modality based medical image retrieval systems. First, owing to the high modality diversity, a single algorithm cannot be capable to differentiate from medical archives^10^. Secondly, precise and absence of resilient dataset is challenging for the development and evaluation of an automated modality classification system. Hence, there is a vital need of an automatic and reliable system which effectively retrieves modality images from medical archives for disease diagnosis and rehabilitation process.

*ImageCLEF* is established for standardization of modality collection and classification^11^. *CLEF Initiative* labs provides an assessment campaign for organizing medical image modality classification challenges. The standard target of *ImageCLEF* is to support the domain of visual media examination, retrieval, and characterization. It works on imperative frameworks for appraisal of visual information recovery system that functioning in monolingual and cross-language settings. The modality classification task was initially presented in *ImageCLEF-2010* campaign, where the total number of modalities limited to eight. Subsequently, data was extended to 18 and 31 in 2011 and 2012 respectively. The *ImageCLEF* campaign is helpful to enhance the retrieval and classification tasks.

**Figure 1:**
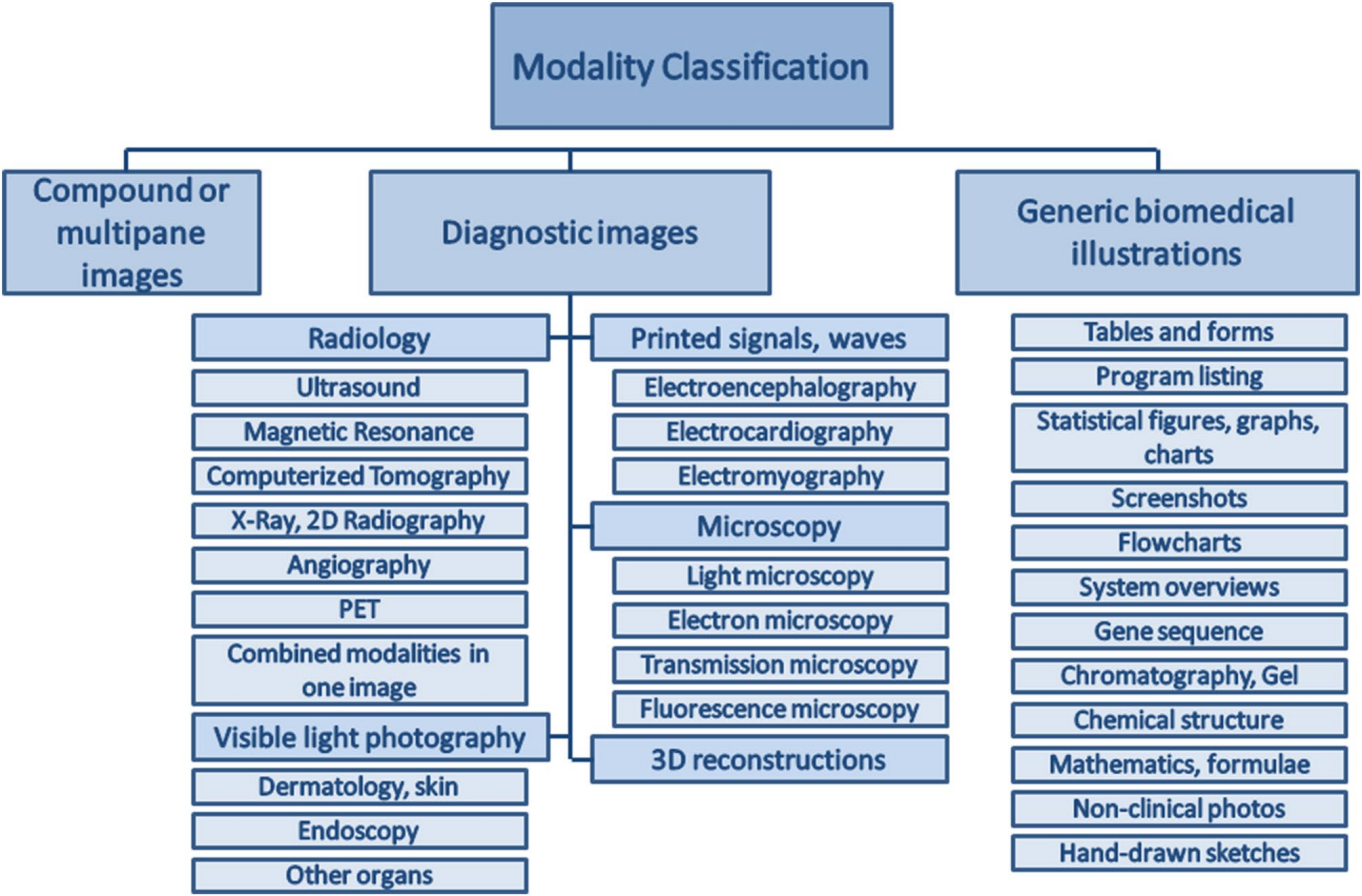
Hierarchy of 31 modalities of *ImageCLEF-2012* dataset^12^.

The main objective of this research is to classify the medical images efficiently and accurately by exploiting the Deep TL features followed by linear discriminant analysis (LDA). For the development of modality classification models, we employed the challenging *ImageCLEF-2012* medical image dataset^12,13^. For training and evaluation of the developed models, *ImageCLEF* has provided annotated dataset. Figure 1 shows the hierarchy of the 31 classes of *ImageCLEF-2012* dataset.

## Related work

Prime purpose of modality classification is to segregate different types of medical images, for instance, X-ray, CT, ultrasound, PET, or general graphs from different medical repositories for disease diagnosis. Effective classification system for retrieval of related clinical cases is required to radiologist for accurate disease diagnosis^14–16^. Development of medical image modality classification system is helpful to limit the retrieval search space^17^ for particular modality. Generally, two main approaches are used for development of modality classification systems: (i) hand-crafted (conventional), and (ii) Deep neural networks.

### Hand-crafted feature based approaches

Unlike general images, medical images have various aspects such as posture complexities, texture and visual features. Medical image modality classification is mainly based on shape, color and texture features^18^. Several researchers have proposed modality classification approaches with the objective to obtain improved performance on benchmark datasets which is developed by *ImageCLEF* organization^19^.

Muller et al. presented an overview of *ImageCLEF-2012* image retrieval task for the 9th edition of *ImageCLEF*^12^. The task was divided into three sub-categories, including modality classification, image, and case-based classification. The aim of competition was to develop a system for precise retrieval of images from large archives that will help the radiologists to retrieve clinical cases efficiently for disease diagnosis and analysis.

Cramer et al*.* proposed a modality classification framework using gray level and color features on the *CISMeF* and the *ImageCLEF-2006* datasets^20^. The proposed framework was applied on two different algorithms for classification of color and gray-scale images. Performance of the developed models decreased as the number of classes increased. Moreover, these models are computationally expensive for classification of color and gray-scale images. Wei et al*.* performed experiments using three different feature extraction techniques, edge histogram descriptor (EHD), Tamura, and Gabor features^21–24^. The developed models provided an accuracy of 60.0% on *ImageCLEF-2012* modality classification dataset.

Khachane et al*.* reported medical image modality classification methodology using SVM and KNN classifiers with fuzzy rule-based techniques^25^. They conducted experiments on five types of modalities including CT, X-ray, Ultrasound, MRI and Microscopic images. The develop models are computationally expensive and their performance decreases significantly as the modality increases.

Arias et al*.* presented Bayesian networks classifiers (BNCs) based hierarchical classification approach using *ImageCLEF-2013* dataset^26^. They performed experiments and obtained 69.21% accuracy and placed third in the competition. They obtained improved classification results due to use of discrete instances by BNCs.

IBM T.J. Watson research group adopted two strategies for modality classification: (i) by augmenting the training examples, and (ii) exploring various features extraction techniques^27^. They constructed combination of seven sets of features for experimentation using different level of granularities. They reported maximum accuracy of 69.9% by performing augmentation on *ImageCLEF-2012* dataset for modality classification competition. Dimitroviski et al*.* evaluated several types of visual and textual features on *ImageCLEF*-2012 modality classification dataset^28^. They have observed the outperformance of SIFT features based classification.

Kitanovski et al*.* proposed modality classification models by combining SVM and *Chi-square* kernel^29^. Similarly, Markonis et al*.*, presented KNN based classification approach^30^. Both approaches utilized different textual, statistical and visual features. They faced the issue of high dimensionality on presenting more features during the model development phase.

Several other researchers have also used similar hand-crafted features for modality classification. Herra et al*.* extracted several features such as SIFT, bag-of color for modality classification^31^. In another study, Pelka et al. proposed modality classification technique utilizing multi-class SVM by combining image features^32,33^. Similarly, Valavanis et al*.* extracted visual, color, edge features used for classification^34^.

It is summarized that the performance of the previously developed approaches using hand-crafted features is varying and achieved overall a sufficient accuracy. This is due to that classification accuracy is highly dependent upon expert assessment while extracting suitable features for modality classification. For efficient classification, it is difficult to guess the numbers and types of extracted features from modality images. These approaches have inherent limitation of high computational requirements and curse of dimensionality. Hence, there is a need to develop an efficient modality classification approach which improves the performance with less human intervention requirements.

### Deep neural networks based approaches

Convolutional neural networks (CNN) are class of deep neural networks introduced by *Fukushima*^35^. LeCun et al. provided improved version of CNN architecture which is being used successfully to solve diverse types of classification problems in computer vision and disease diagnosis^36^. CNN have an ability to learn image features automatically by utilizing a set of several non-linear transformations. For efficient classification, it has capability to extract most important features at different stages of abstraction.

In literature, several Deep learning models are proposed for classification and customization. These models are extensively being applied and validated on benchmark *ImageNet* dataset of 1,000 classes^37^. A number of CNN architectures are developed for *ImageNet* challenge. Krizhevsky et al*.* have developed *AlexNet* classification model of CNN architecture and reported the landmark breakthrough utilizing the GPU^38^. Szegady et al*.* developed *GoogLeNe*t Deep learning model to reduce the problem of over-fitting^39^. They have achieved improved performance by reducing neural networks parameters compared to the *AlexNet*. He et al*.* introduced Deep Residual Network (*ResNet*) architecture to solve the degradation problem of model training^40^.

Generally, during the development of CNN models, a large amount of annotated dataset is required. In medical domain, data labeling is challenging and time consuming process to get adequate labeled dataset for CNN model development. To overcome this issue, transfer learning (TL) concept in Deep learning is introduced. TL utilizes the existing state-of-the-art Deep learning trained models for classification^41,42^. The pre-trained model parameters are updated based on the customized (new) dataset. Researches are using TL concept along with fine-tuning of existing CNN models for medical image classification and disease detection^43,44^.

Rajpurkar et al*.* developed (*CheXNet*) Deep learning model to detect fourteen types of chest pneumonia disease using X-ray images^45^. They obtained detection rate at the level of radiologists with reduced human efforts. In another study, Gulshan et al*.* applied Deep learning model for diabetic detection using retinal fundus images^46^. Similarly, Esteva et al*.* proposed CNN image based model for skin cancer detection and successfully classified the disease^47^. Anthimopoulos et al*.* developed CNN based system for inter statistical-lung disease detection and classification into seven classes (one out of seven is healthy) and reported an accuracy of 85.5%^48^. Similarly, Tudler and Bruijne developed restricted *Boltz* machine and CNN based models for lungs disease classification^49^. Moeskop et al*.*, used CNN for segmentation of brain MR images^50^. They developed models using extracting multiple patches of various sizes. Yan et al*.*^51^ proposed a multi-instances CNN models for body-part recognition in CT and MR images. The approach comprised of two phases: (i) train the model on discriminative patches (ii) fine-tuned network was tested for twelve classes.

Kumar et al*.* developed models using *AleNet*^38^ and *GoogLeNet*^39^ to make an ensemble of features by employing the TL concept for modality classification^52^. They performed classification using SVM and attained an accuracy of 84.3% on *ImageCLEF-2016.* Similarly, Yu et al*.*^53^ obtained an accuracy of 87.8% using TL concept for medical image modality classification on *ImageCLEF-2016* dataset.

In this study, we utilized challenging *ImageCLEF-2012* dataset for development of modality classification system. This dataset is highly skewed and having diverse types of 31 classes. It is hard to identify and extract discriminant hand-crafted features to build improved performance system. In this research, we employed Deep TL concept and fine-tuned the pre-trained model for construction of medical image modality classification system^54^. For optimal performance, Deep features are exploited by the LDA classifier. The developed approach provided excellent performance on *ImageCLEF-2012* multi-class classification problem.

## Results

Extensive experiments are performed to evaluate and validate the usefulness of the developed approach. The approach is applied to classify multiclass problem using benchmark *ImageCLEF-2012* medical image modality dataset. Details of dataset can be found in material section. We obtained Deep TL *ResNet50* features to develop classification model in combination with LDA (*TLRN-LDA*) algorithm. Performance of the developed approach is compared with state-of-the-art approaches and hand-crafted features based classification using overall and class-wise measures. The effectiveness of the develop model is also evaluated by comparing with TL *ResNet50* using softmax classifier.

Table 1 shows an average performance measures of the proposed approach in terms of accuracy, sensitivity, specificity, precision, F-score, and MCC are 0.88, 0.89, 0.99, 0.88, 0.88 and 0.88, respectively. From Table 1, it is inferred that each performance measure accomplished high average value more than 88%. This high performance indicates the usefulness of the developed approach for modality classification.

**Table 1:** Performance of the TLRN-LDA on benchmark *ImageCLEF-2012* dataset.

Performance measure	Average value (%)
Accuracy	87.91
Sensitivity	89.30
Specificity	99.60
F-Score	88.30
Precision	88.00
MCC	88.10

**Figure 2:**
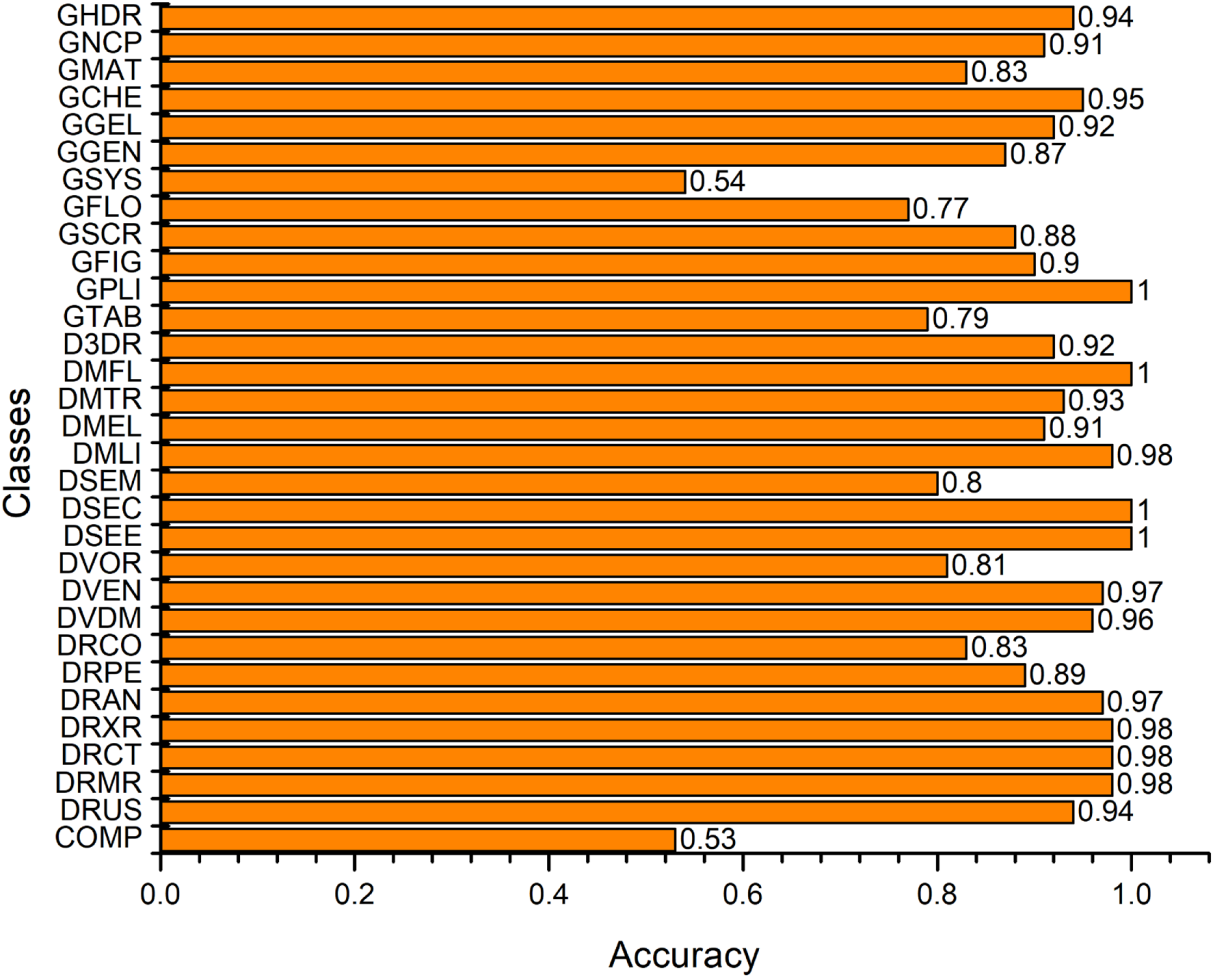
Class-wise accuracy of the proposed *TLRN-LDA*.

**Figure 3:**
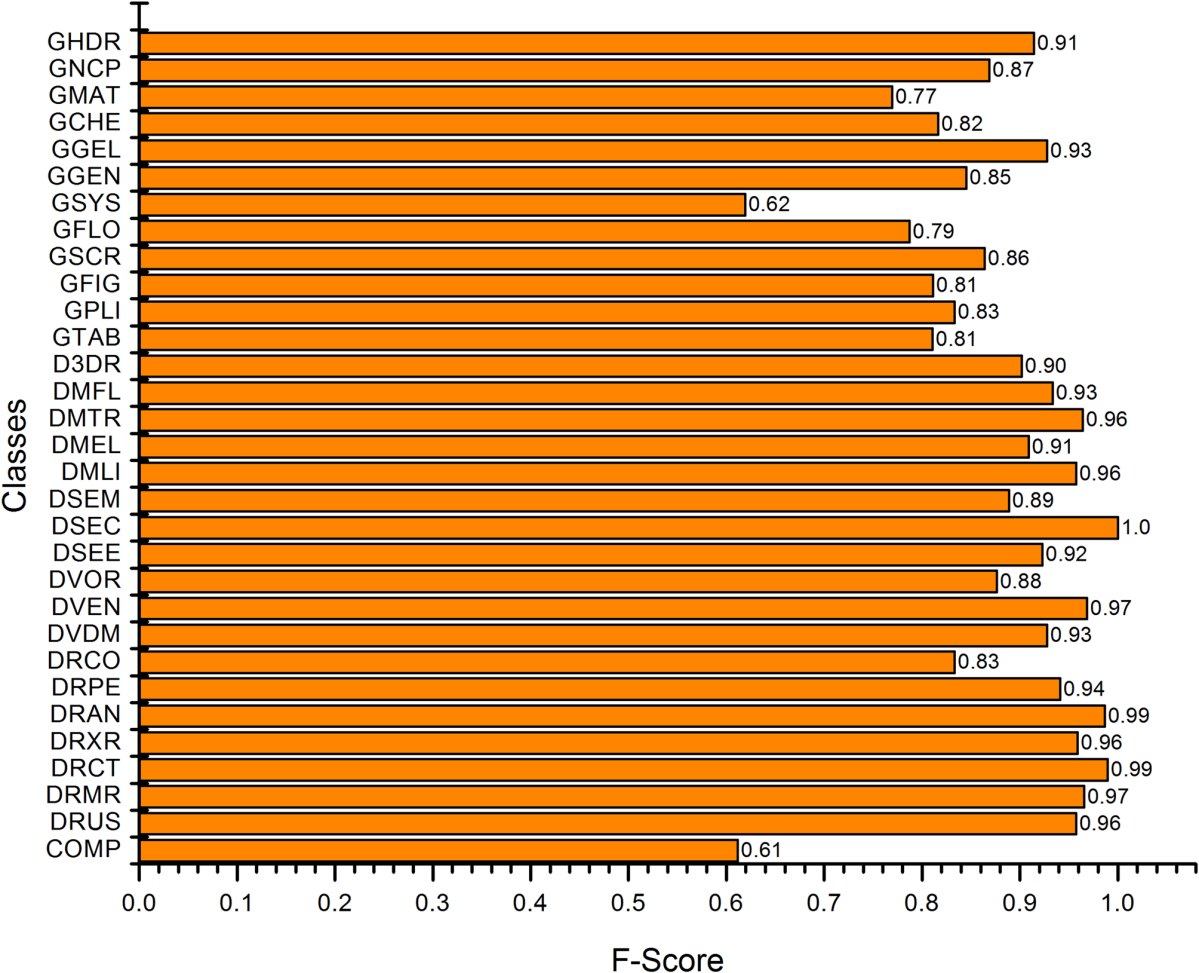
Class-wise F-score of the proposed *TLRN-LDA*.

In multiclass problem, class-wise performance evaluation is inevitable. Figure 2 depicts class-wise accuracy of the proposed approach. From Fig. 2, it is observed that our model achieved the maximum and minimum class accuracies of 100% and 53%, respectively. Overall, most of the classes attained greater than 80% accuracy. However, classes ‘COMP’, and ‘GSYS’ provided an accuracy lower than average. Figure 3 shows class-wise F-score of the proposed model which successfully identified the positive classes with high precision.

Training performance in terms of accuracy, sensitivity, specificity, precision, F-score, MCC, and class-wise accuracy of the developed approach is shown in supplementary material Figures S1, S2, and S3. Accuracy and loss for training and testing of *TL-ResNet50* at different iterations are shown in Figure S4 and S5, respectively.

**Figure 4:**
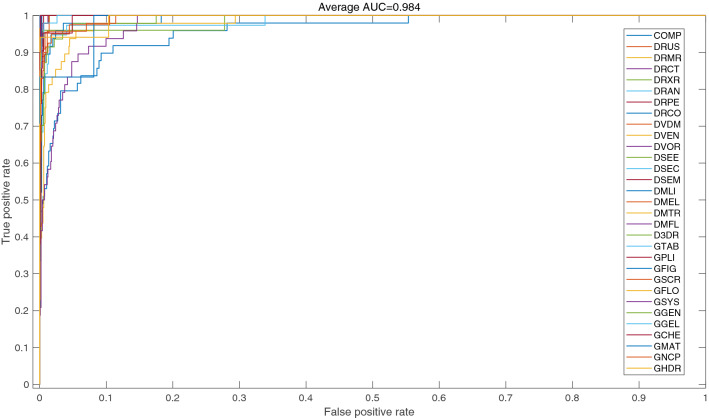
ROC curves of the *TLRN-LDA* for 31 classes.

For accurate disease diagnosis, radiologist needs high true positive rate (TPR) and low false positive rate (FPR). ROC curve is one of the effective measures that simultaneously provide knowledge about TPR and FPR. Class-wise ROC curves are shown in Fig. 4. It is observed that most of the class curves are aligned with vertical axis and provided maximum AUC except classes ‘COMP’, and ‘GSYS’. This trend is similar to class-wise accuracy which is shown in Fig. 2. The proposed approach attained excellent performance in terms of average AUC (98.4%).

**Figure 5:**
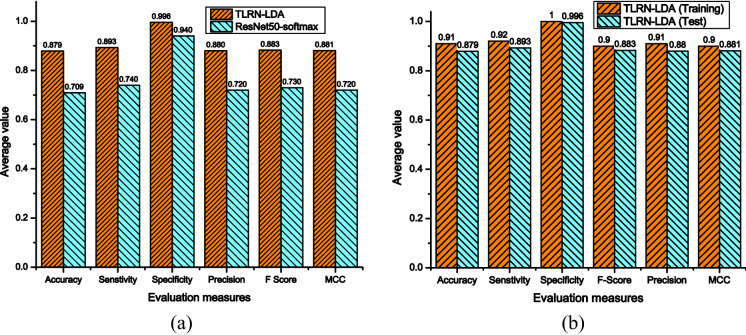
Performance comparison of the proposed approach (**a**) *TLRN-LDA* training and testing and (**b**) *TLRN-LDA* and *ResNet50*-softmax.

The performance comparison of the developed *TLRN-LDA* and TL *ResNet50*-softmax classification is illustrated in Fig. 5a. It is observed from Fig. 5a that our approach demonstrated sufficient enhancement in each performance measure. For instance, accuracy is improved by 19% over TL *ResNet50*-*softmax*. The improved performance highlights that the proposed approach effectively learns the challenging multiclass *ImageCLEF-2012* dataset. For fair comparison, we have included training and testing performance of the *TLRN-LDA* in Fig. 5b.

Table 2 shows performance comparison of the developed approach with state-of-the-art approaches on the same benchmark *ImageCLEF-2012* dataset. The proposed approach achieved high accuracy of 87.9%. Whereas, visual and texture feature based SVM classification model offers a maximum accuracy of 78.6%. Our approach improved accuracy of 16.5% over the visual and texture based SVM model. To validate the effectiveness of the developed approach, we extracted several hand-crafted features such as SIFT, LBP, LTP, EHD, CEDD, color edge detector using wavelet transform and color histogram, and developed hybrid features based LDA classification model. It provided 71.4% accuracy which is 16.5% lower than *TLRN-LDA*. Again, performance of our approach outperformed over hand-crafted feature based model.

**Table 2:** Performance comparison of the developed approach with state-of-the-art approaches on *ImageCLEF-2012* dataset.

Approach	Description	Accuracy (%)
IBM multimedia analytics^27^	Data augmentation, visual features and SVM classification	69.60
Dimitrovski et al.^28^	Several visual and textual features and SVM classification	78.60
*medGIFT*^31^	Mixed type of hand crafted features	66.20
Present study		
Hybrid feature set	Mixed hand-crafted features of SIFT, BOW, LBP, LTP, HOG, ECD, ECDWT and LDA classification	71.4
TLRN-LDA	*ResNet50* transfer learning Deep features and LDA classification	87.91

## Discussion

For accurate disease diagnosis, medical image modality classification is one of the challenging tasks to retrieve the related clinical cases from large medical repository. Practitioner can get benefit for better diagnostic, treatment and rehabilitation decisions through the use of modern retrieval technologies. In this regard, we developed an intelligent and effective modality classification approach which offers excellent performance. Usually, Deep learning technique requires large amount of annotated datasets for training and validation of a model. On the other hand, medical data is inherently available in small amount. For this, we exploited the learning capability of Deep learning with TL concept to develop the modality classification models. The developed approach offers improved performance and has capability to perform high classification on relatively small image modality dataset. Unlike traditional CNN architectures, the proposed approach was developed by combining the TL *ResNet50* feature space with LDA technique. In LDA, the objective function of Eq. (5) optimizes the ratio of between and within-class variances and thereby guaranteed the maximum class separability. The linear combinations which maximize Eq. (5) produce low variance of the same class and high variance for different classes in the projected space. In LDA model development process, each class is considered as a separate class against all others (one vs all) and obtained merely one lower dimensional space for other classes to project their data on it. In this way, we exploited the useful properties of LDA such as low intra-class variance, high inter-class variance, and optimal decision boundaries. It has achieved improved performance compared to TL *ResNet50-softmax* for medical image modality classification. The proposed approach mainly composed of two main parts: (i) construction of TL *ResNet50* model for optimal Deep feature extraction and (ii) development of LDA classifier.

The original residual network (*ResNet)* architecture with 50 layers (*ResNet50*) has been trained on standard *ImageNet* dataset having 1,000 classes. This dataset does not contain classes related to our problem i.e. medical image modality (*ImageCLEF-2012*). Misclassification is expected, if *ImageCLEF2012* dataset is directly feed to *ResNet50* originally trained on general images which do not have any medical image modality classes*.* To overcome this issue, we used TL concept in conjunction with *ResNet50* to classify *ImageCLEF-2012* dataset of 31 classes.

**Figure 6:**
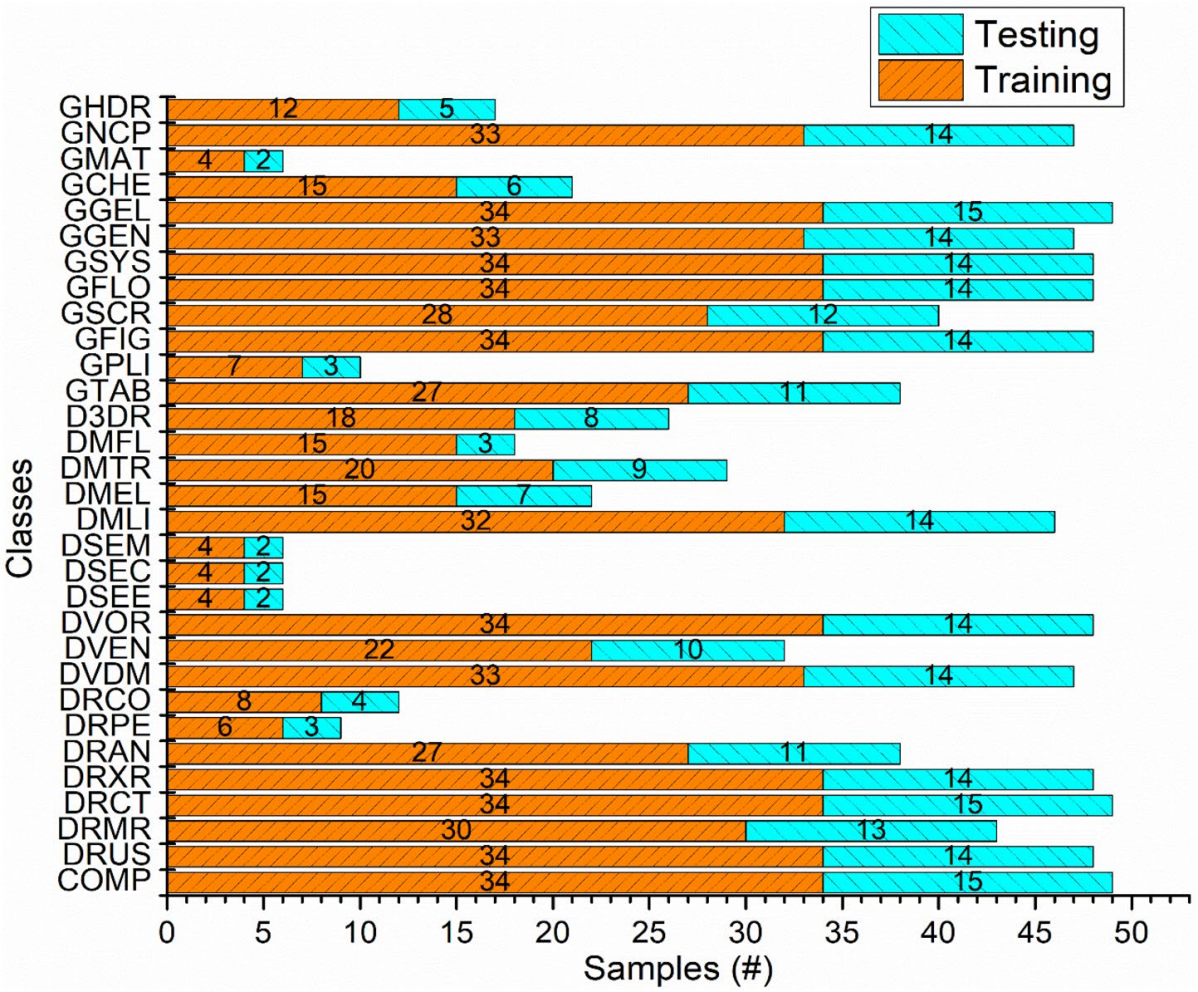
Class-wise distribution of *ImgeCLEF-2102* dataset. Each class sample is randomly divided into training and testing of ratio 70%:30%, respectively.

Analysis of Fig. 6 shows that the *ImageCLEF-2012* dataset used in this study have five classes with less than ten samples whereas other classes have relatively more samples thus the dataset is highly skewed. Generally, it is assumed that classification models offer sufficient performance on classes having more training instances. From Figs. 2 and 6, it is observed that developed approach attained high classification accuracy of 100%, 98%, 100%, 83%, and 97% for ‘DSEC’, ‘DSEM’, ‘DSEE’, ‘GMAT’ and ‘DRPE’ classes, having relatively less data samples, respectively. On the other hand, ‘COMP’ and ‘GSYS’ classes have higher data samples of 49 and 48 respectively but for both classes developed approach provided low class-wise accuracy of 53% and 54%, respectively.

It is evident that during the modeling process, performance of the model is increased with relatively smaller training instances while decreased with larger training instances. Owing to the high diversity of images in the dataset for ‘COMP’ and ‘GSYS’ classes, performance of the developed model is decreased. On the other hand, *TLRN-LDA* efficiently learned the patterns and offer high performance on those classes with small number of instances. It is observed that small data sample classes with high inter and low intra-class variances played helpful role in the model development process. On the other hand, relatively more misclassification occurs for those classes which having large data sample with high intra-class variance. Figures 3 and 4 also supported to this fact in terms of F-score, and ROC curves measures, respectively. It is evident from the ROC curves that the classes with lower accuracy attained less AUC. The model may offer sufficiently high performance, if one can overcome the intra-class variation.

The comparison of training (Supplementary Figs. S1, S2, and S3) and testing (Figs. 2, 3, 5) performance shows the similar trend of the developed models. It is observed that training and testing models provided low performance on classes ‘COMP’, and ‘GSYS’ which having high intra-class variances.

From Table 1, it is observed that on employing TL concept the proposed approach achieved improved classification accuracy of 87.9%. This indicates that the model successfully learned the features and weights on new dataset. Obviously, the residual learning capability of *ResNet50* helps in obtaining optimal Deep features. The FC layer of *ResNet50* is replaced and networks learned the weights according to *ImageCLEF-2012* dataset of 31 classes. Deep features are obtained at ‘*avg_pool*’ layer of the *ResNet50* model. These learned features are exploited for development of LDA classification model. To validate the effectiveness, performance comparison of the developed model with *ResNet50-softmax* is shown in Fig. 5. It revealed that our approach offers improved performance in terms of average accuracy, sensitivity, specificity, precision, F-score, and MCC by 18%, 15%, 5%, 16%, 15%, and 16% over the *ResNet50-softmax* model. The develop LDA classifier achieved better performance because it successfully explored the patterns of Deep features for small dataset whereas, *ResNet50-softmax* requires large amount of data for search space exploration.

The enhanced performance, in terms of accuracy (87.9%) of the developed approach is revealed compared to state of the approaches on the same *ImageCLEF-2012* dataset^27,28,31^ (Table 2). Cao et al*.,* from IBM multimedia analytics group was the winner of the *ImageCLEF-2012* competition^27^. They reported 69.9% classification accuracy by performing data augmentation for class balancing, extracted several visual image features followed by SVM classification. Thus, in terms of accuracy, an improvement of 18.31% is evident compared to Cao et al*.* approach^27^. Dimitrovski et al*.* reported 78.6% accuracy on *ImageCLEF-2012* dataset by extracting visual and textual feature fusion followed by SVM classification^28^. Thus, an improvement of 9.31% is found compared to Dimitrovski et al. model^28^. Herrera et al*.* from *medGIFT* group extracted several visual hand crafted features and remains the runner-up and reported 66.2% classification accuracy on the same dataset^31^. It is observed that in terms of accuracy, an improvement of 21.71% is found compared to Herrera et al.^31^ model^31^. Further, from Table 2, it is inferred that an improvement of 21.71% in performance of the developed approached compared to hybrid hand crafted features based classification (present study).

The approaches compared in Table 2 used same input *ImageCLEF-2012* dataset (1,001 images) for development of models. However, our approach is differing with the compared approaches in two ways: (i) we used LDA classifier for final classification instead of softmax and (ii) splitting of training and testing datasets for model development and evaluation. In first case, we employed LDA classifier on Deep features for final classification that is helpful for obtaining improved image modality classification. In other case, to obtain better generalization, we subdivided the dataset (1,001 images) into two parts (70% training, 30% test) and then fivefold cross-validation data resampling technique is applied. The relationship between training and testing results indicate better generalization of the develop model.

The developed approach outperformed over previously developed hand craft feature based approaches. The superior performance of our approach is might be due to (i) extraction of optimal Deep image features at ‘*avg_pool*’ layer of *ResNet50* (ii) better generalization of the developed model and (iii) utilization of LDA classifier for better discrimination.

Owing to the skewed nature of *ImageCLEF-2012* dataset it is challenging to perform classification with high accuracy without any data augmentation. TL and fine-tuning employed on *ResNet50* provided most optimal and discriminate features for the development of LDA classification. This high classification performance of the developed approach revealed that *ResNet50* is successfully learned the medical image modality features and LDA classifier better learned inter-class and intra-class variances than softmax classification at relatively small dataset. Values of the evaluated measures indicated the effectiveness of the developed model. The proposed approach can be used as a tool for modality classification by the radiologists or information retrieval products.

Our developed framework could be implemented on new daily data. Moreover, in future, we are intending to deploy the developed system in hospital for daily use of modality image retrieval for disease diagnosis by radiologists. The implemented computer codes are available for downloading at our University site for modifications and experimentations. Now, *ImageCLEF* has diverted its tasks from modality classification to seperate combined figures in *ImageCLEF-2015* and onward datasets. In other word, tasks for the newer version of *ImageCLEF* dataset are changed from independent modality classification to sub-figure separation instead of independent modality classification. However, in our future research plan, we are intending to implement the developed framework with modifications for the most recent data from *ImageCLEF* for automatic image captioning and scene understanding, medical visual question answering and decision support on tuberculosis.

## Conclusions and future directions

In this research, we introduced a novel approach to explore the potential of TL concept on pre-trained Deep learning model in conjunction with LDA for development of medical image modality classification system. TL concept and fine-tuning of *ResNet50* to extract the Deep features. These features are exploited to develop LDA classifier for image modality classification. The developed approach has achieved high classification accuracy of 87.9% on the benchmark *ImageCELF-2102* dataset. It has offered improved performance on skewed as well as high inter-class variation in the dataset. The performance of the developed approach depends on useful information extracted from TL *ResNet50* and better capability of the LDA classifier to capture inter-class and intra-class patterns of relatively small datasets. It has demonstrated robustness for independent testing dataset. Overall, the proposed approach improved classification accuracy of 21.71% over the state-of-the-art approaches on *ImageCLEF-2012* dataset. The proposed approach attained 18% and 17% improvement in terms of accuracy compared to *ResNet50-softmax* and hybrid handcrafted features based LDA classification models, respectively. Comparative analysis highlighted that our approach offers superior performance over the conventional approaches. This outstanding performance is due to optimal learned features which are obtained from TL *ResNet50* followed by LDA classification. It is anticipated that our study would be helpful for information retrieval technologies, particularly for disease diagnosis and treatment decisions. In the future, this approach can be extended to develop multi-level classification system for the diagnosis of modality-specific diseases.

## Materials and method

### Materials

In Deep learning there are two main categories: (i) design a new network architecture using large amount of annotated data and (ii) model development using TL concept with relatively small amount of data. TL concept becomes popular to solve a new classification problem especially at small data. In this research, TL concept has been employed on pre-trained *ResNet50* model and fine-tuned on benchmark *ImageCLEF-2012* dataset for modality classification^54^. The benchmark material obtained from *ImageCLEF* for the development of Deep TL features followed by the LDA for modality classification. Details of dataset and its class-wise distribution are described below.

### Dataset

The *ImageCLEF-2012* dataset developed by the *ImageCLEF* organization used for medical image modality classification^19^. The *ImageCELF-2012* dataset was obtained from the datasets of several thousand biomedical articles. These articles were retrieved from *PubMed Central (ncbi.nlm.nih.gov/pmc*/) using open access journals that permitted free redistribution of the data. The dataset is freely available for download under *Creative Common License* by signing end user agreement.

We performed experiments for modality classification on benchmark *ImageCELF-2012* dataset, which consists of 1,001 images with 31 modalities. Figure 6 shows class-wise distribution along with training and testing samples of the modalities of the *ImageCLEF-2012* dataset. This dataset contains images of various sizes and is highly skewed in nature. Splitting of original dataset samples for training and testing may vary but ratio will remain same. It is observed that five classes have less than 10 samples. In this scenario, it is challenging to develop an efficient classification model. We developed a Deep TL based model using LDA classifier without any data augmentation to obtain high classification performance.

### Methods

**Figure 7:**
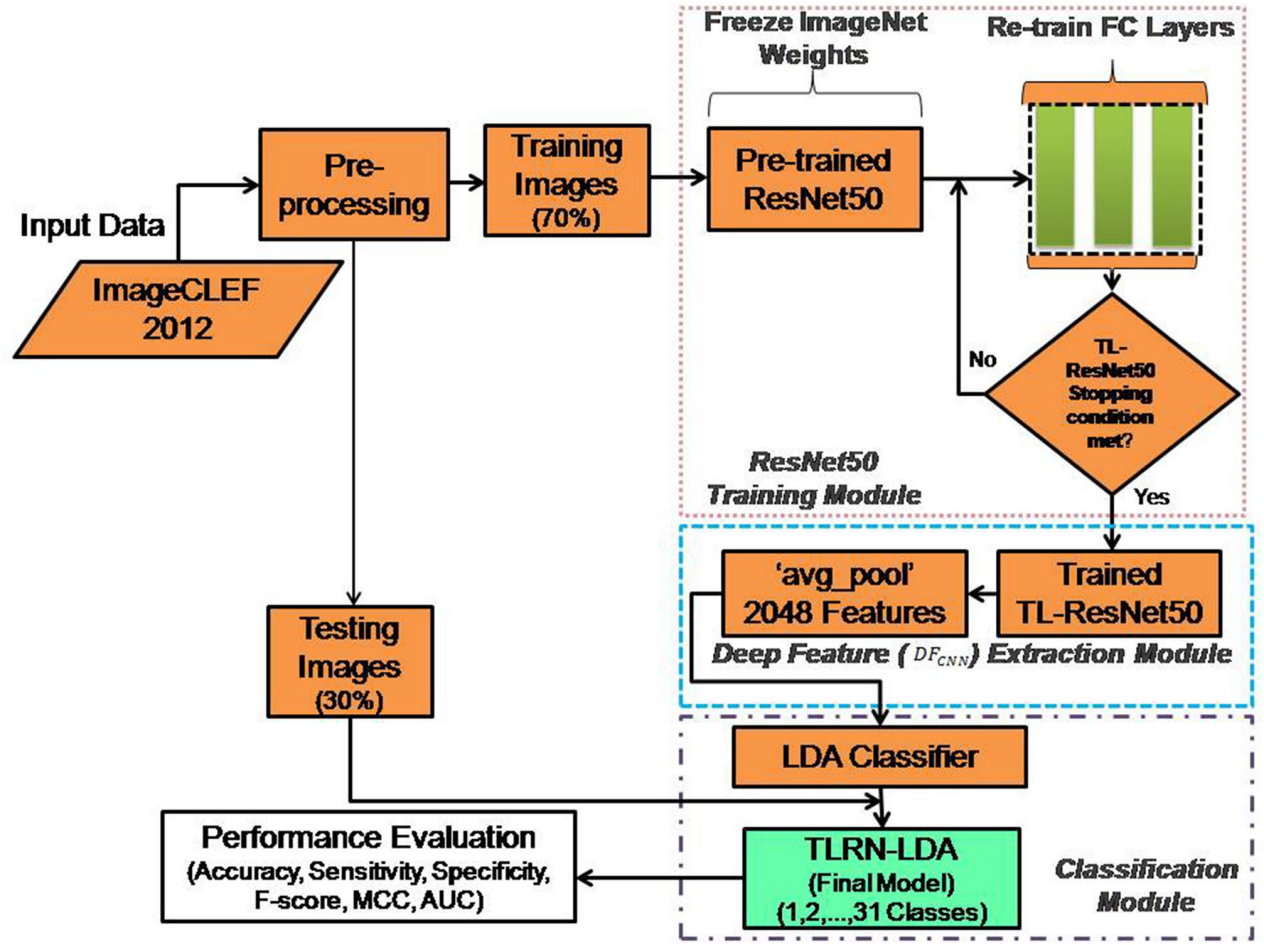
Framework of the proposed *TLRN-LDA* system for medical image modality classification.

The framework of the proposed approach for modality classification is shown in Fig. 7. The dataset of *ImageCLEF-2012* is randomly divided into 70:30% proportion for model training and testing, respectively. The proposed approach is comprised of two parts: (i) training of TL*-ResNet50* for Deep feature extraction followed by LDA classification and (ii) testing of the developed model. In part-I, the final model “*TLRN-LDA*” is developed on 70% training dataset using fivefold cross-validation data resampling technique. In this cross-validation technique fourfolds are used for training and remaining fold is employed for validation. Then average classification performance of the model is computed. This process provides generalization results of the trained (*TLRN-LDA*) model. In this way around 5 h training time is required to obtain the final model.

The training part consists of three modules. In first module, TL based pre-trained ResNet50 model is trained on 70% training dataset. In second module, Deep features are extracted at ‘*avg_pool*’ layer just before the FC layers of *ResNet50* model^40^. In this way, a feature vector of size 2048 is obtained for each modality image. In third module, LDA classification model is developed using Deep features. LDA utilizes an optimized classification criterion to exploit inter and intra-class variances. Finally, training performance of the developed model is computed. After successful training, the *TLRN-LDA* model has been evaluated on independent 30% unseen dataset which is not used during model training and development. The detailed description of the proposed approach is given in the following sub-sections.

### TL-*ResNet50* training and feature extraction modules

The input dimension of *ResNet50* network is 224 × 224 × 3. First of all, we perform pre-processing step to resize *ImageCLEF-2012* dataset images and align it with *ResNet50* network input size. In the TL *ResNet50* training module, we obtained pre-trained *ResNet50* model and employed TL concept on the last fully connect (FC) layers (FC1000, FC1000_softmax and classificationlayer_FC1000). Originally, the *ResNet50* was trained on *ImageNet* dataset of 1,000 classes^37^. The pre-trained *ImageNet* weights except the last FC layers of *ResNet50* are frozen and utilized to develop model for modality classification problem. It is appropriate to use TL concept on pre-trained Deep learning model on relatively small dataset, instead of training the model from scratch on which requires very large dataset. In this way, we trained the model to learn weights of FC layers for medical image modality classification. This modified TL *ResNet50* network is now trained and fine tuned on new dataset of 31 classes instead of 1,000.

**Figure 8:**
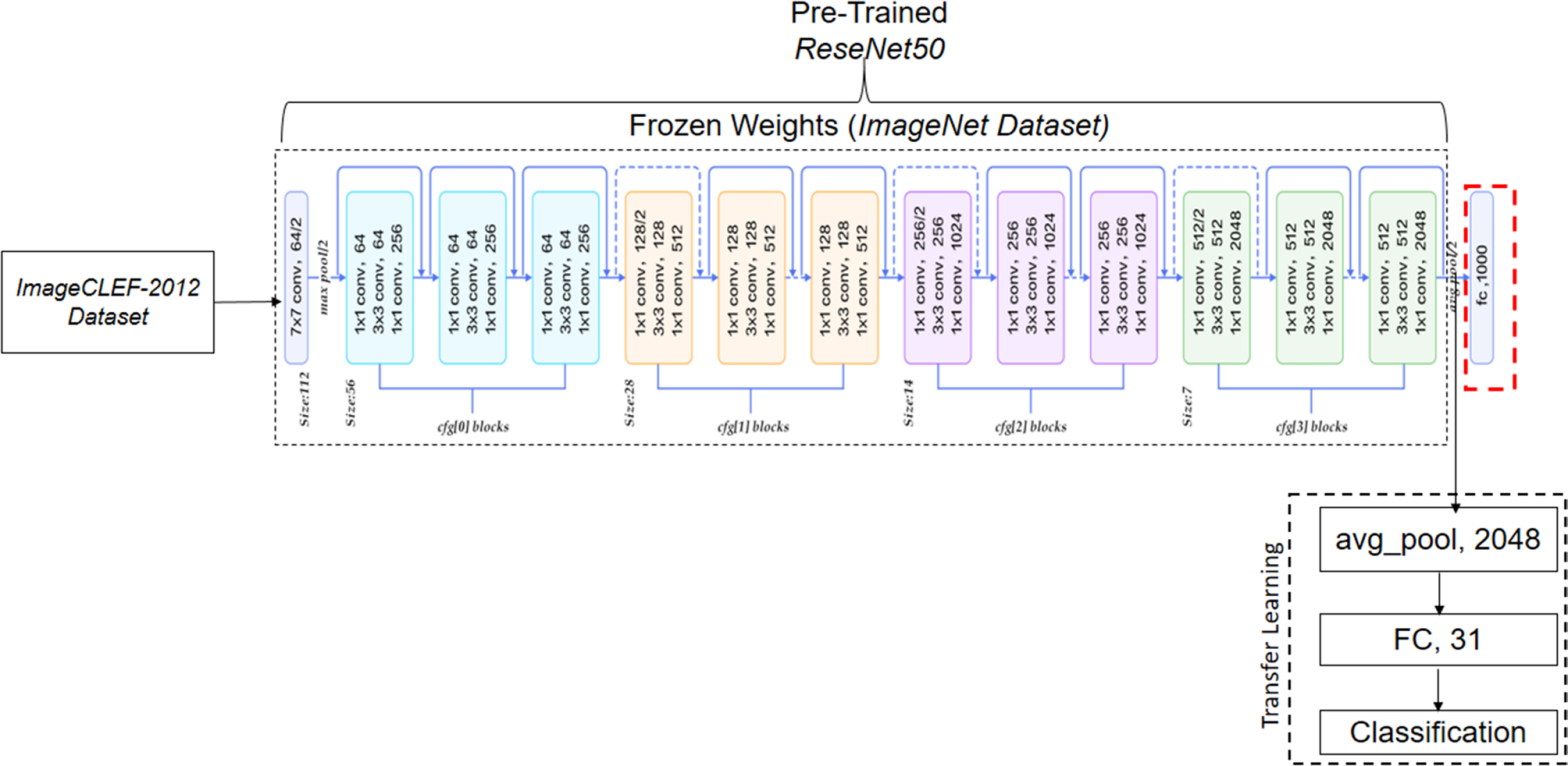
The proposed TL *ResNet50* architecture for modality classification.

In Deep feature extraction module, TL *ResNet50* network is trained on *ImageCLEF-2012* dataset to extract Deep feature on *‘avg_pool’* layer before last FC layers of *ResNet50* model as shown in Fig. 8. The TL *ResNet50* works as an arbitrary feature extractor, it allows the new input image to propagate forward and stopped at pre-defined layer (‘*avg_pool’*) to obtain Deep features. By freezing the pre-trained *ImageNet* weights, we can still exploit the robustness and discriminative learning capability of TL *ResNet50*. An optimal Deep feature vector of size 2048 has been obtained at ‘*avg_pool’* layer by employing TL concept for modality classification. The obtained high classification performance Deep features are feed to LDA for final classification.

In the original network, FC layer of *ResNet50* provides an optimal features followed by softmax classification. In this research, we employed TL *ResNet50* for modality classification. Figure 8 shows the replacement of fully connected layers FC1000, FC1000_softmax and classificationlayer_FC1000 of *ResNet50* with FC31, FC31_softmax, and classificationlayer_FC31, respectively. Additionally, pre-trained *ResNet50* model weights prior to FC layers are frozen.

**Figure 9:**
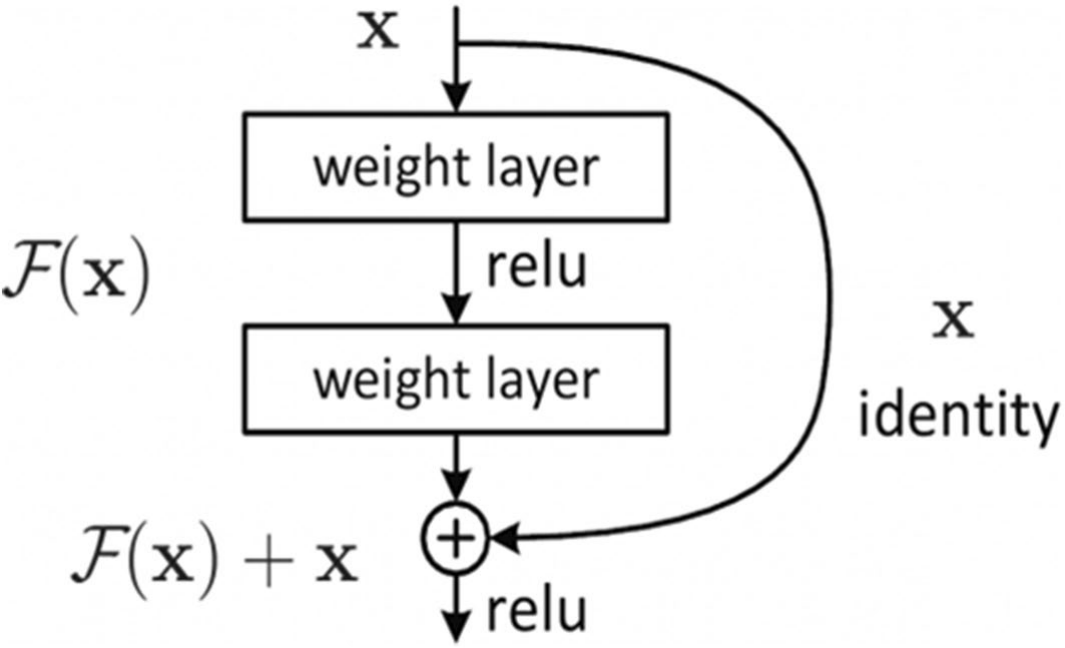
Residual learning process of *ResNet50*^40^.

For better parameter optimization and higher performance, *ResNet50* utilized the concept of residual learning compared to other Deep learning architectures such as *VGG*^55^ and *AlexNet*^38^. The concept of residual learning is shown in Fig. 9. When activation function *F*(*x*) returns zero, it bypasses the block with an identity mapping $y = x$, where $x$ represents the input to the layer. The residual learning process reduces overfitting and offers generalized classification models. Detail of *ResNet50* can be found in^40^.

Back-propagation approach is employed on $X = \{ x_{1} ,x_{2} , \ldots ,x_{n} \}$ training dataset to learn the weights of last three FC layers. The objective function Eq. (1) is minimized to learn optimal weights $W_{CNN}$ on new training dataset.

1 $$loss\left( {W_{CNN} ,\;X} \right) = \frac{1}{n}\mathop \sum \limits_{i = 1}^{n} l\left( {f\left( {x_{i} , \;w_{CNN} } \right),c_{i} } \right)$$

where, $x$ is a feature vector corresponding to input image of the dataset, $f$ is prediction function, $w_{CNN}$ are learned weights and $c$ is actual class label. Function $l$ computes loss on training sets.

Gradient decent function is used to obtain optimal weights. The training process stopped by reaching 700 epochs. We set the learning rate (0.0005) and the momentum (0.9) empirically. Total training time of TL *ResNet50* was 5 h and 20 min. The proposed approach is implemented on Matlab 2018(9.5b) on Intel Core i7 16 GB RAM and NVIDIA GeForece GTX 105 for CNN model training and validation.

### Development of LDA modality classification module

Generally, CNN architectures are widely used softmax for classification^38–40^. *ResNet50* also uses softmax a generic form of logistic regression. Owing to the small and skewed nature of *ImageCLEF-2012* dataset, we applied LDA instead of softmax classifier. However, for comparison purpose, we have also obtained results of same dataset on softmax classification. Several studies indicated the usefulness of the LDA for classification. Wu et al*.*^56^ performs experiments for person re-identification using LDA. In another study, Pohar et al*.*^57^ reported that the performance of LDA for multi-class problem and observed that its performance is better compared to logistic regression (LR). They analyzed that in case of more than five classes, LDA outperformed over softmax. To the best of our knowledge, in the context of medical image modality classification, LDA is not exploited on Deep features.

In this module, TL *ResNet50* Deep features dataset,${\mathbf{DF}}_{{{\mathbf{CNN}}}} = \left\{ {{\mathbf{df}}_{1} ,{\mathbf{df}}_{2} , \ldots ,{\mathbf{df}}_{{\mathbf{n}}} } \right\},$ is labeled for 31 classes, where “**df**_**1**_” represents data of Deep features of class 1 and “**df**_**2**_” represents data of Deep features of class 2 and so forth. Each dataset has “n” variables and there are “m” such datasets out of which “m_1_” belong to class 1 and “m_2_” belong to class 2 and so on. In other words, “${\mathbf{DF}}_{{{\mathbf{CNN}}}}$” is a data matrix with size “m × n”, “**df**_**1**_” is a data matrix with size “m_1_ × n” and “**df**_**2**_” is a data matrix with size “m_2_ × n”, and so on. For LDA training, the dataset of Deep features is randomly partitioned into the ratio of 70:30% for model training and testing respectively. The fivefold cross validation technique is used to develop LDA model.

LDA applied on the extracted Deep features ${\varvec{DF}}_{{{\varvec{CNN}}}}$ to get LD score matrix ${\varvec{Z}}$.

2 $${\varvec{Z}} = {\varvec{DF}}_{{{\varvec{CNN}}}} \times {\varvec{W}}$$

The basic objective is to find linear combination which optimally divides our multiclass annotations into discriminant groups. LDA algorithm tries to search the weight vector, ${\varvec{W}} = \{ w_{1} ,w_{2} , \ldots ,w_{l} \}$, where *l *is the number of solutions, which maximizes the rate between and within-class scatters. Defining the between-class scatter $CS_{bc}$.

3 $$CS_{bc} = \mathop \sum \limits_{i = 1}^{C} (\mu_{i} - \mu )(\mu_{i} - \mu )^{T}$$

and within-class scatter $CS_{wc}$

4 $$CS_{wc} = \mathop \sum \limits_{i = 1}^{c} \mathop \sum \limits_{j = 1}^{{m_{j} }} (\mu_{j} - \mu_{i} )(\mu_{j} - \mu_{i} )^{T}$$

where $\mu_{i}$ denotes mean of class *i*,$m_{j}$ denotes total number of observations of the *i*th class,$\mu_{j}$ is one such observation and *T* denotes the transpose.

The objective function $J({\varvec{W}})$ is defined using Eqs. (3) and (4) as:

5 $$J\left( {\varvec{W}} \right) = \frac{{{\varvec{W}}^{T} CS_{bc } {\varvec{W}}}}{{{\varvec{W}}^{T } CS_{wc } {\varvec{W}}}}$$

Find weight vector ${\varvec{W}}^{*}$, which associated to variable on discriminant function, such that $J$ is maximized. The resulting LD scores matrix “**Z**” represents compactly the original data features, “${\mathbf{DF}}_{{{\mathbf{CNN}}}}$” and differentiates one class from another very efficiently. Detail of LDA can be found in^58–60^.

### *TLRN-LDA* testing

Testing module of the developed model is very straightforward. In this module, the input image is pre-processed and fed to the trained TL *ResNet50* for feature extraction followed by trained LDA classification (*TLRN-LDA*). Label of input medical modality image is predicted by the trained LDA and compared with the actual label as shown in classification module of proposed framework (Fig. 7).

### Performance evaluation

The developed model is assessed using standard classification performance evaluation measures such as accuracy, sensitivity, specificity, F-score, Mathew correlation coefficient (MCC), ROC curves and area under the curve (AUC). These measures are frequently used for evaluation of classification models. Performance of the proposed model is compared with other state-of-the-art approaches on the *ImageCLEF-2012* dataset. It is observed that the proposed system offers superior results compared to its competitors.

### Informed consent

Informed consent was obtained from all individual participants included in the study (https://www.imageclef.org/).

## Supplementary information

Supplementary Figures.
