# Supplementary material for: Developing intelligent medical image modality classification system using deep transfer learning and LDA
Source: Sci Rep. 2020 Jul 30;10:12868. doi: 10.1038/s41598-020-69813-2 (PMC7393510; doi:10.1038/s41598-020-69813-2)
Supplement: Supplementary file 1 — Supplementary Figures. [file 41598_2020_69813_MOESM1_ESM.pdf]

Developing intelligent medical image modality classification system using deep transfer learning and LDA

Mehdi Hassan[1*], Safdar Ali[2], Hani Alquhayz[3], Khushbakht Safdar[4]

[1]Department of Computer Science, Air University, PAF Complex Sector E-9, Islamabad Pakistan
[2] Directorate General National Repository, Islmabad Paksitan
[3]Department of Computer Science and Information, College of Science in Zulfi, Majmaah University, Al-Majmaah, 11952, Saudi Arabia
[4]Al Nafees Medical College and Teaching Hospital, ISRA University, Lehtrar Road,Islamabad Pakistan

*Corresponding Author:

Mehdi Hassan

Tel: +92-51-9263335-7, Fax: +92-51-9260158

mehdi.hassan@mail.au.edu.pk , mehdi.hassan5@gmail.com

Supplementary Material

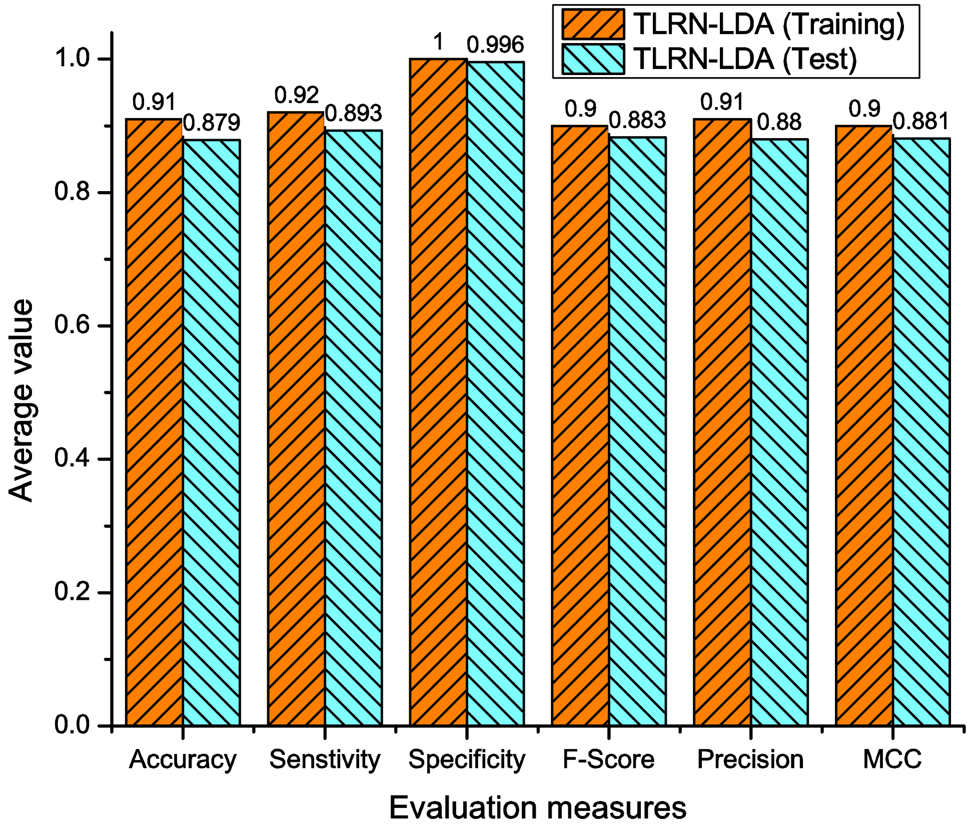

Figure S1: Training and testing performance comparison of the TLRN-LDA model.

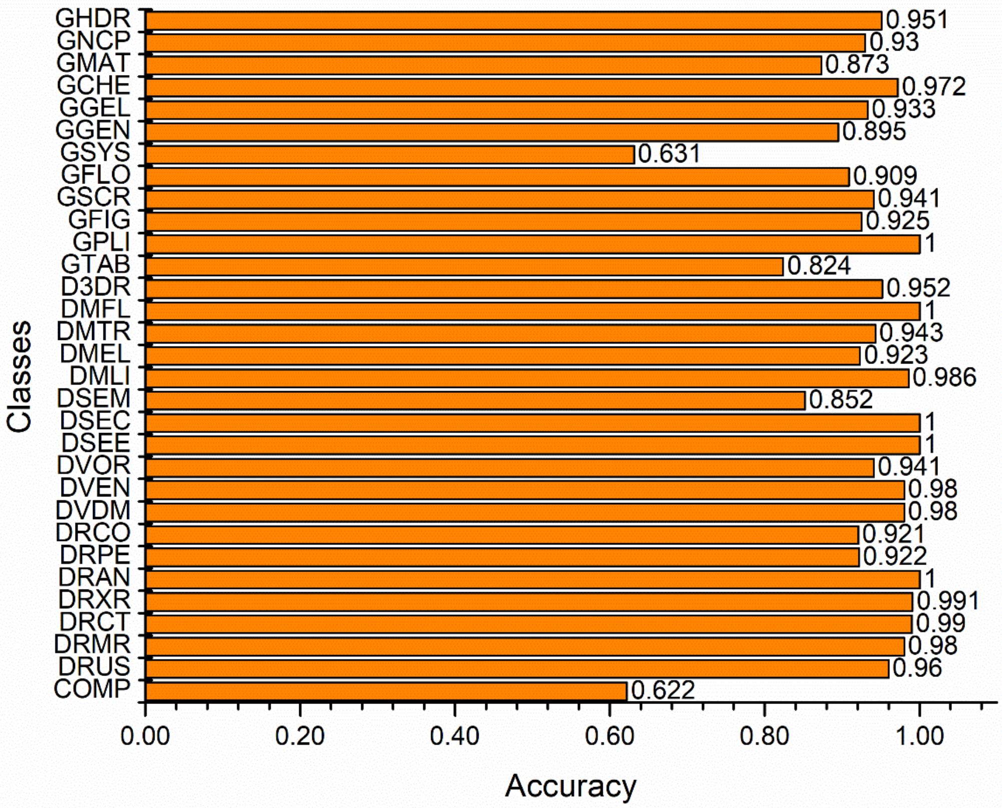

Figure S2: Class-wise training accuracy of TLRN-LDA

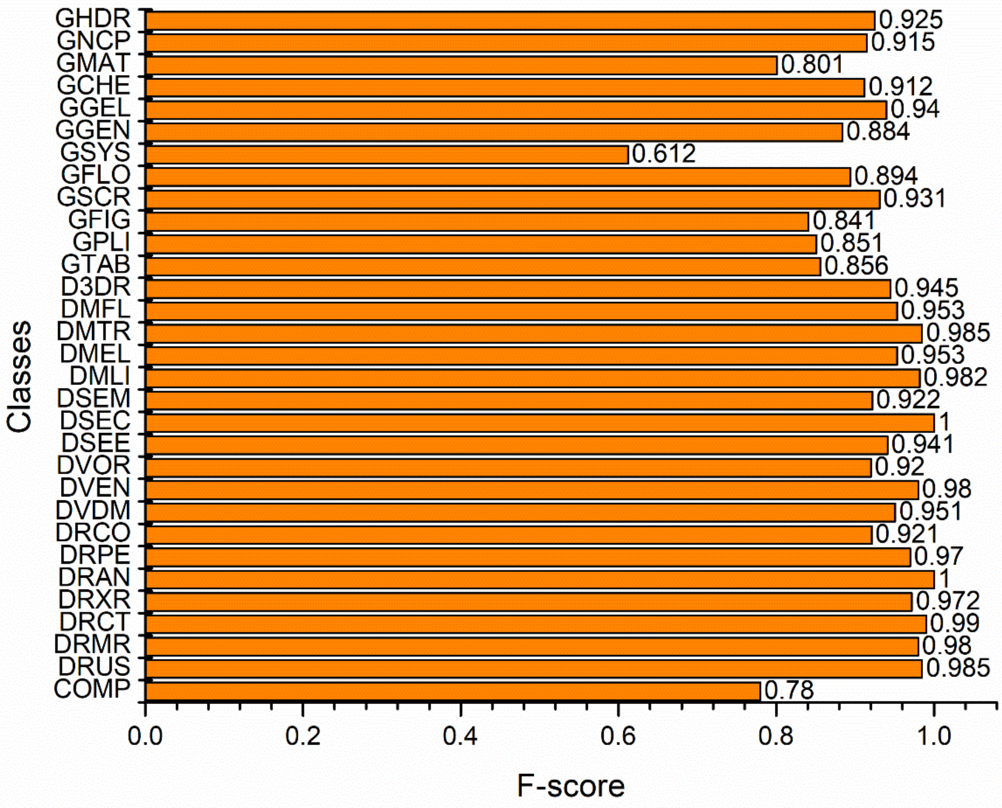

Figure S3: Class-wise training F-score measure of TLRN-LDA

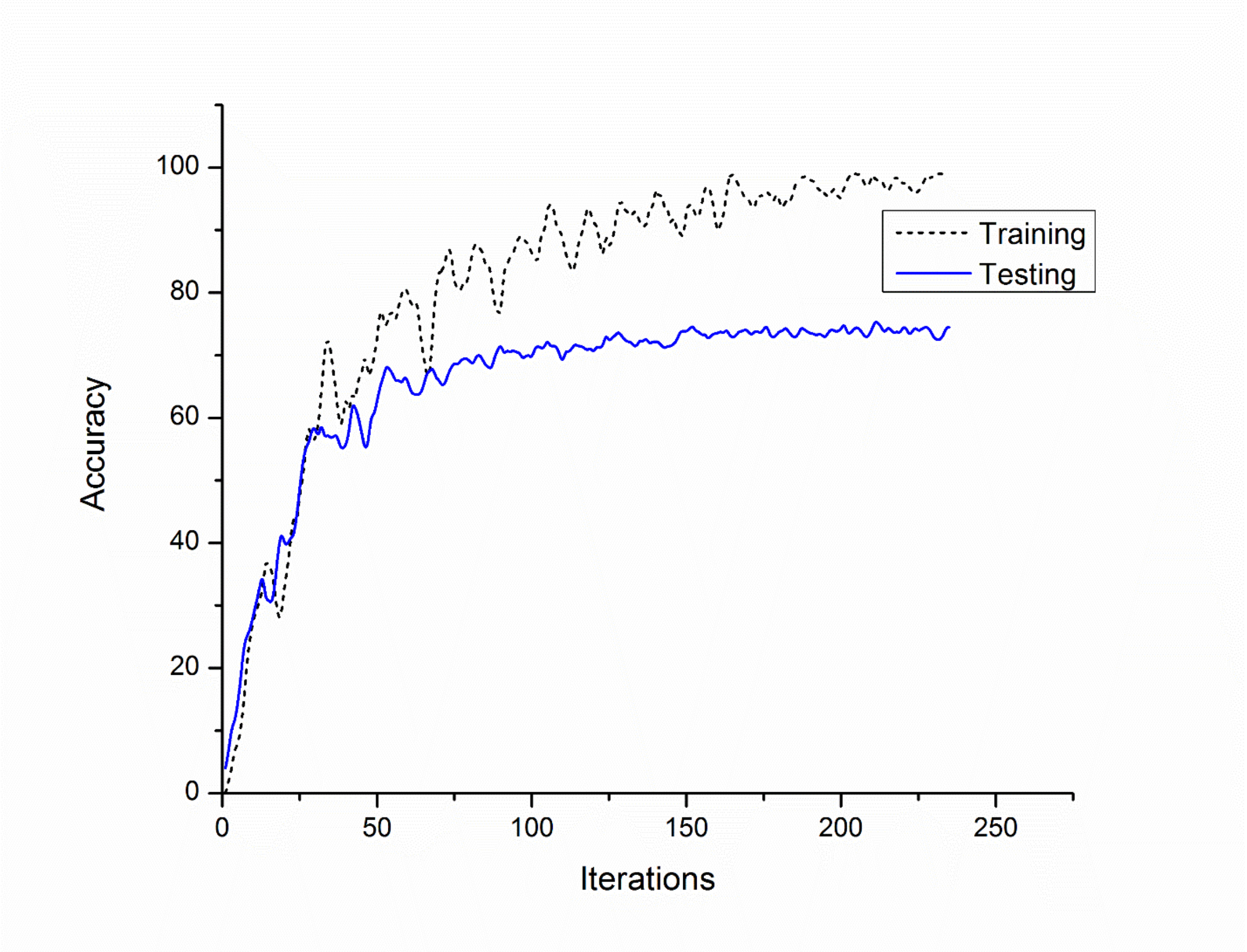

Figure S4: Training and testing accuracy of TL-ResNet50 at different iterations

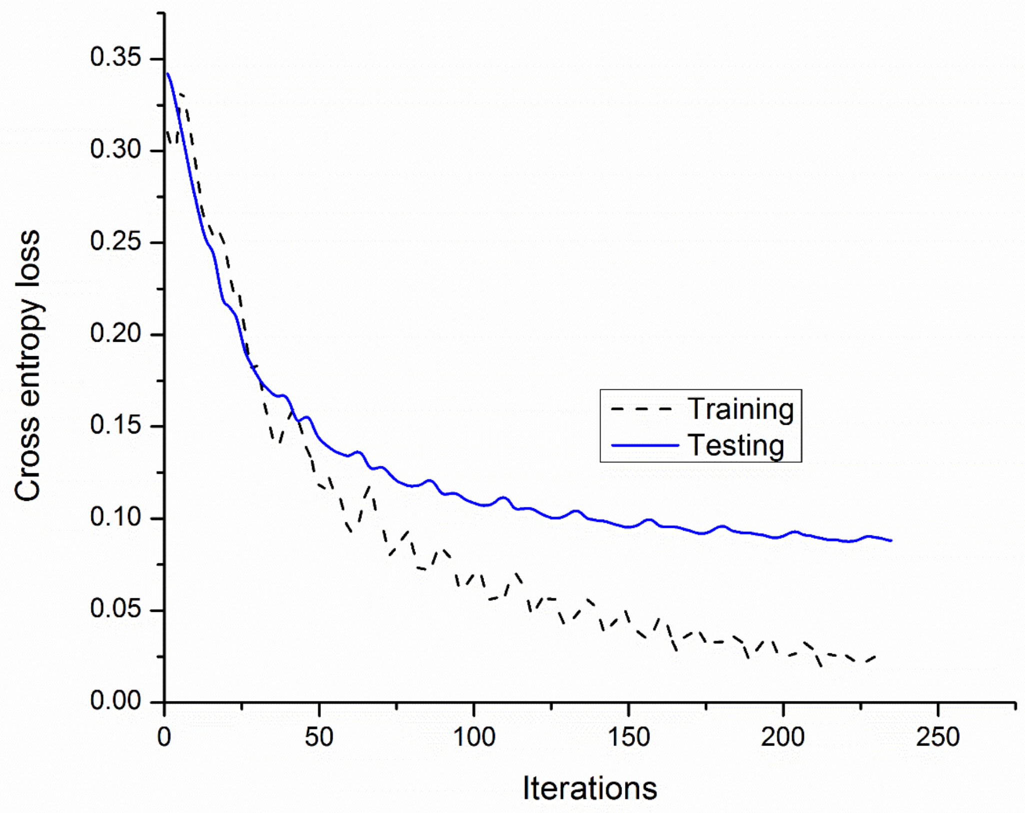

Figure S5: Training and testing loss of TL-ResNet50 at different iterations
